# Complete genomes of *Pasteurella multocida* isolates from porcine, bovine, and cervine samples found in New Zealand farms

**DOI:** 10.1128/MRA.00884-23

**Published:** 2023-11-22

**Authors:** Ruy Jauregui, Andrea Barcelo, Peter Bennett, Jonathan Foxwell, Kelly Buckle, John O'Connell, Bede Busby, Michelle McCulley

**Affiliations:** 1 Animal Health Laboratory, Biosecurity New Zealand, Ministry for Primary Industries, Upper Hutt, New Zealand; University of Maryland School of Medicine, Baltimore, Maryland, USA

**Keywords:** pasteurella, farm, zoonosis

## Abstract

Here, we present complete genome assemblies of *Pasteurella multocida* strains isolated from porcine, bovine, and cervine farms as part of bacteriology incursion investigations to identify pathogens that might present a sanitary risk to New Zealand.

## ANNOUNCEMENT

*Pasteurella multocida*, the causative agent of the zoonotic disease pasteurellosis, is a bacterium of economic relevance to the livestock industry in New Zealand. Pasteurella colonizes a broad spectrum of animal hosts, including pigs, cattle, and deer.

Initial identification of *Pasteurella multocida* was performed on cultures isolated on blood agar with 5% sheep blood (Fort Richard Laboratories, Auckland, New Zealand) grown for 24 h at 37°C. Samples labeled W22-3735-1, W22-00979-1, and W22-2894-7 were identified using MALDI-ToF Mass Spectrometry (Bruker MALDI Biotyper^TM^, confidence scores 2.00–2.14), and sample W16-01607-1 was identified using capsular typing PCR. Isolates were confirmed as *P. multocida* using a PCR adapted from reference (1) with the KMT1-SP6/KMT1 T7 primers. The capsular typing of each isolate was characterized using a multiplex PCR adapted from reference (2). Exotic-to-New Zealand hemorrhagic septicemia-causing (HS) strains and toxA toxin-producing strains were ruled out using PCRs adapted from references (1) (KT-T72/KT-SP61 primer set) and (3), respectively.

DNA for whole-genome sequencing was extracted using a QIAGEN DNA mini kit, and concentration was quantified using the Qubit dsDNA HS Assay protocol (Thermo Fisher Scientific, New Zealand). DNA library preparation was performed for two platforms: Illumina MiSeq and Oxford Nanopore Technologies (ONT) MinION.

DNA extraction quality was assessed using the Agilent TapeStation 4200 with the HS DNA ScreenTape D5000 protocol. In the case of the MiSeq, library preparation was performed using the Illumina DNA Prep kit and Nextera DNA CD indices, as recommended by the manufacturer. Sequencing was performed on a MiSeq platform following Illumina’s protocol for 300 nt pair-end sequencing with the MiSeq Reagent kit v2 with 500 cycles. Library preparation for ONT MinION was performed using the Rapid gDNA Barcoding SQK-RBK114.24 protocol. After assessing the quality of the libraries, a single pooled library was loaded into the flow cell with chemistry R9.4.1 following the manufacturers’ protocol.

All programs were run with default parameters unless otherwise noted. Illumina fastq reads were quality-inspected using fastQC v0.11.9 (4) and trimmed using Trimmomatic v0.39 (5) to remove Nextera adapters and trim bases with average quality <15 in a sliding window of five nucleotides. Nanopore reads were base-called with Guppy v6.5.7 and model dna_r10.3_450bps_hac, quality-inspected using nanoQC v0.90.4 (6), and trimmed using Chopper v0.5.0 (6) to remove low-quality tailends (50 nt from the head and 20 nt from the tail).

Reads were hybrid assembled with Unicycler v0.5.0 (7), including circularization and rotation steps. Genome completeness was evaluated using BUSCO v5.4.7 (8) and the bacterial database odb_10. The sequence type was obtained using the program Krokus v1.0.3 (9) and the pubMLST (10) databases for *Pasteurella multocida*. Taxonomy classification was verified with TYGS (11), showing *Pasteurella multocida* type species ATCC 43137 as the closest relative. Genome assemblies were annotated at submission time using the NCBI Prokaryotic Genome Annotation Pipeline (PGAP) v6.6. Table 1 compiles read and assemblies statistics.

**TABLE 1:** Sample and read data, and genome assembly stats

Data set	Feature	Isolates			
		W22-3735.1	W22-00979.1	W16-01607.1	W22-2894-7
	Host	Bovine	Cervine	Cervine	Porcine
	Origin	Peritoneal fluid	Lung tissue	Sinus abscess swab	Lung swab
Illumina	Read pair no.	2598206	1765612	1855543	2097018
	Coverage	680	477	477	522
	SRA link	SRR25497972	SRR25497971	SRR25497970	SRR25497969
Nanopore	Read no.	1885539	689187	455274	148459
	Coverage	124	88	159	226
	N50/L50	239539/3.27Kb	65084/7.82Kb	62811/17.34Kb	21061/7.26Kb
	SRA link	SRR25497968	SRR25497967	SRR25497966	SRR25497965
Assembly	Contig no.	1	1	1	1
	Length	2.29 Mb	2.27 Mb	2.3 Mb	2.3 Mb
	GC%	40%	40%	40%	40%
	BUSCO score	99.1%	99.1%	99.2%	99.2%
	GenBank link	CP133810	CP133809	CP133808	CP133807
Annotation	Gene no.	2135	2143	2163	2162
	tRNA no.	57	57	58	58
	rRNA no.	19	19	19	19
Sequence typing	MLST multi-host	ND^*a*^	ND	161	New
	MLST RIRDC	62	64	179	New

^*a*^ ND, not determined.

These samples were collected by local district investigators as part of clinical/diagnostic surveillance, precluding requirements for animal ethics approval.

## Data Availability

Genomes have been deposited in GenBank and Raw reads in the SRA. Table 1 presents links to the publicly available data.
